# Non-malarial febrile illness: a systematic review of published aetiological studies and case reports from Southern Asia and South-eastern Asia, 1980–2015

**DOI:** 10.1186/s12916-020-01745-0

**Published:** 2020-09-21

**Authors:** Poojan Shrestha, Prabin Dahal, Chinwe Ogbonnaa-Njoku, Debashish Das, Kasia Stepniewska, Nigel V. Thomas, Heidi Hopkins, John A. Crump, David Bell, Paul N. Newton, Elizabeth A. Ashley, Philippe J. Guérin

**Affiliations:** 1 Infectious Diseases Data Observatory (IDDO), University of Oxford, NDMRB, Old Road Campus, Oxford, OX3 7FZ UK; 2 Centre for Tropical Medicine and Global Health, Nuffield Department of Clinical Medicine, University of Oxford, Oxford, UK; 3 London School of Hygiene and Tropical Medicine, London, WC1E 7HT UK; 4 Centre for International Health, University of Otago, Dunedin, New Zealand; 5 Issaquah, USA; 6 Lao-Oxford-Mahosot Hospital-Wellcome Trust Research Unit, Vientiane, Laos

**Keywords:** Fever, Aetiology, Febrile illness, Malaria, Southern Asia, South-eastern Asia, Diagnostic

## Abstract

**Background:**

In the absence of definitive diagnosis, healthcare providers are likely to prescribe empirical antibacterials to those who test negative for malaria. This problem is of critical importance in Southern Asia (SA) and South-eastern Asia (SEA) where high levels of antimicrobial consumption and high prevalence of antimicrobial resistance have been reported. To improve management and guide further diagnostic test development, better understanding is needed of the true causative agents of fever and their geographical variability.

**Methods:**

We conducted a systematic review of published literature (1980–2015) to characterise the spectrum of pathogens causing non-malarial febrile illness in SA and SEA. We searched six databases in English and French languages: MEDLINE, EMBASE, Global Health (CABI) database, WHO Global Health Library, PASCAL, and Bulletin de la Société Française de Parasitologie (BDSP). Selection criteria included reporting on an infection or infections with a confirmed diagnosis, defined as pathogens detected in or cultured from samples from normally sterile sites, or serological evidence of current or past infection.

**Results:**

A total of 29,558 records from 19 countries in SA and SEA were screened, of which 2410 (8.1%) met the selection criteria. Bacterial aetiologies were reported in 1235 (51.2%) articles, viral in 846 (35.1%), parasitic in 132 (5.5%), and fungal in 54 (2.2%), and 143 (6.0%) articles reported more than one pathogen group. In descending order of frequency, *Salmonella* Typhi, *Escherichia coli*, *Staphylococcus aureus*, *Klebsiella pneumoniae*, and coagulase negative *Staphylococcus* were the commonly reported bacteria, while dengue virus, chikungunya virus, Japanese encephalitis virus, hepatitis B virus, and hepatitis C virus were common viral pathogens reported. Reports of rarely reported or emerging pathogens included a case report of *Borrelia burgdorferi* (Lyme disease) in India in 2010 and reports of Nipah virus in Singapore and India.

**Conclusions:**

This review summarises the reported non-malaria pathogens that may cause febrile illness in SA and SEA. The findings emphasise the need of standardising the reporting of aetiological studies to develop effective, evidence-based fever management and improved surveillance. Research and development of diagnostic tools would benefit from up-to-date epidemiological reporting of the regional diversities of non-malaria fever aetiologies.

**Trial registration:**

PROSPERO registration, CRD42016049281

## Background

While a global decline in malaria burden has been reported in the last 15 years, Asia has observed the sharpest decrease [1, 2]. The most recognised cause of febrile illnesses has gradually shifted from malaria to other infectious diseases in Southern Asia (SA) and South-eastern Asia (SEA) [3, 4]. In some endemic parts of Asia, as little as 1% of febrile illnesses among those visiting healthcare facilities has been attributed to malaria [5, 6]. In these areas, once malaria is ruled out from the differential diagnosis, delineating the cause of febrile illness can be challenging. Contributing factors include the limited availability of rapid diagnostic tests (RDTs) for infections other than malaria and limited microbiology laboratory facilities for the identification of the microorganisms, many of which require skilled personnel as well as relatively complex and expensive equipment and reagents [7]. Improving the diagnosis and management of febrile illnesses caused by non-malarial pathogens—referred to henceforth as non-malarial febrile illness (NMFI)—is a regional and global priority [3, 5, 8, 9]. The World Health Organization (WHO) has emphasised the need to identify the pathogens causing NMFIs for the development of country-specific algorithms for effective fever management, especially in primary healthcare facilities [8].

Fever is one of the commonest reasons to seek medical attention in this region [10]. However, there is a paucity of information regarding the geography of aetiological agents of fever for many countries in SA and SEA [4, 11]. With approximately 2.5 billion inhabitants, a third of the world’s population living in SA and SEA, the scarcity of information is of upmost importance to guide public health policies and research and development investments. This region has seen the emergence, re-emergence, and spread of several pathogens of serious threat to global health like dengue, chikungunya, influenza A (H5N1 and H1N1), and different multidrug-resistant infections among many others [12, 13]. For the clinician or a health worker in an outpatient clinic in resource limited settings, the uncertainty in disease diagnosis can prompt indiscriminate use of broad-spectrum antimicrobials including combinations of antibacterials, antiparasitics, and antifungals without prior knowledge of the likely aetiological pathogen or underlying antimicrobial susceptibility. There are concerns that this practice promotes antimicrobial resistance (AMR) [14, 15], while rates of antimicrobial consumption are rising globally [16]. The widespread availability of over-the-counter antimicrobials, self-medication practices, overprescribing, poor information, poor adherence, and the lack of rapid diagnostics to differentiate infections requiring antimicrobials from those that do not are all potential drivers of AMR in this region [15, 17]. At the same time, access to life-saving antimicrobials must be assured for those who need them.

Thus, finding available and pertinent evidence to compensate for the lack of aetiological knowledge of NMFIs will aid in enhancing surveillance strategies, fever diagnostics, and effective fever management and contribute to antimicrobial stewardship efforts in this densely populated region. In line with this, an initial effort was made in 2012 to map the regional distribution and abundance of the pathogens in the Mekong sub-region and this project expands that work [9].

A major challenge for assimilation of available evidence on NMFI is the dearth of epidemiologically sound, methodologically rigorous, and standardised evidence. This precludes us from reliably assessing the distribution of prevalent fever-causing agents in the SA and SEA regions [11, 18]. In light of this sporadic and non-standardised reporting of fever research, we conducted an exhaustive systematic review of all published literature from 1980 through 2015, including evidence meeting minimal selection criteria to be as inclusive as possible in studying the reported distribution of the broad spectrum of pathogens in this region.

## Methods

This review was conducted according to the Preferred Reporting Items for Systematic Reviews and Meta-Analyses (PRISMA) statement [19]. The study protocol is registered with the international prospective register of systematic reviews (PROSPERO Registration ID: CRD42016049281).

### Literature search strategy

A systematic literature search was carried out in six libraries: MEDLINE, EMBASE, WHO Global Health Library (SEARO and WPRO files), Global Health (CABI), Banque de Données Santé Publique (BDSP), and PASCAL to identify publications from 1980 through 2015. A broad search string was employed which consisted of Medical Subject Headings (MeSH) and free text terms with no restriction on study design (Supplemental file 1; section 1.1).

The literature searches for SA and SEA were carried out separately with different time periods used (Supplemental file 1; section 1.1). Restrictions were imposed to exclude articles published before 1980 and those published in languages other than Chinese, English, French, Portuguese, or Spanish. The corresponding author or the journal was contacted to provide articles when necessary.

### Study selection and full-text review

**Fig. 1:**
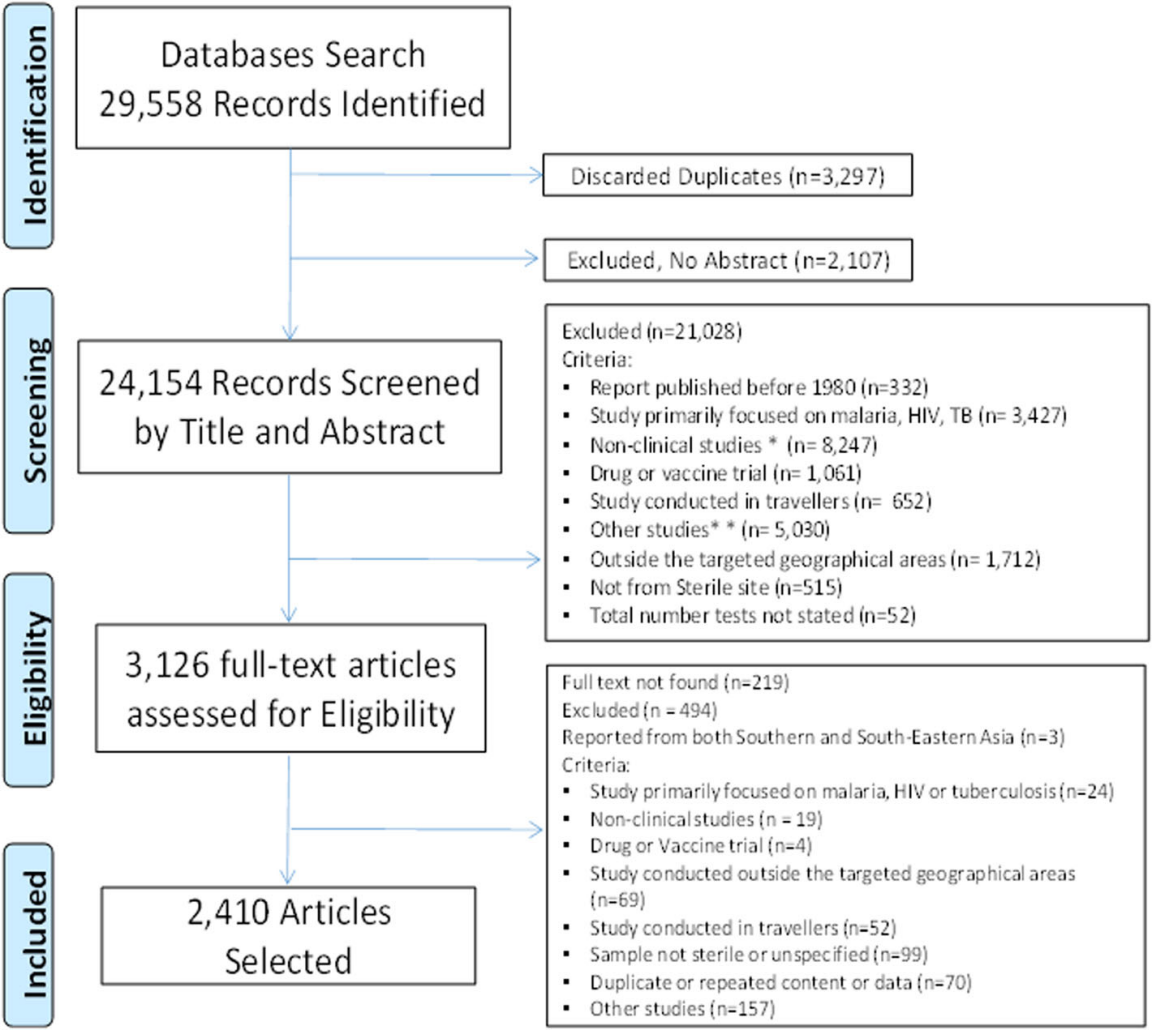
PRISMA flow diagram, a systematic review of publications from Southern Asia and South-eastern Asia, 1980–2015. *Non-clinical studies = descriptions of laboratory methods, modelling studies, economic evaluations, opinion pieces, animal model, and studies on medicinal plants. **Other studies = studies of disease not including laboratory identification of pathogens causing fever (vector transmission, genetic studies, empirical diagnosis)

The screening was carried out in two stages to identify the articles fulfilling the inclusion criteria: title and abstract screening (stage I) and then full-text screening (stage II). Only articles meeting the pre-specified inclusion and exclusion criteria were included. Non-malarial febrile illness: a systematic review of published aetiological studies and case reports from Africa, 1980-2015. Two reviewers assessed the articles for SA (PS) and SEA (CON) independently—with each article assessed only once by one of the two reviewers. The PRISMA flow diagram is presented in Fig. 1, and a separate flow diagram for SA and SEA reviews is shown in Supplemental file 1 (Supplemental file 1; section 1.2).

### Data extraction

Data from articles deemed eligible for the review were extracted into a bespoke online data extraction form built for the purpose of this review. The extracted variables included the following bibliographic metrics: study title, names of the first and second author, year of publication, Uniform Resource Locator (URL), and the digital object identifier (DOI). Information on the following study meta-data was extracted: name of the study site, latitude and longitude of the study site(s), study start year, study end year, and range of age groups included. The type of sample, number of individuals tested, number of cases testing positive for an organism, and laboratory method/s used were recorded for the reported pathogens.

### Study design

Each of the reports identified in the review was classified into one of three groups: (i) case report/case series, (ii) seroprevalence studies, and (iii) fever series. A fever series was defined as studies where the number of participants tested was clearly stated together with the number of participants testing positive for a given pathogen with an accurate diagnostic test for pathogen identification (culture or molecular methods). Seroprevalence studies were defined as studies that reported the number of individuals testing positive for a serological test along with the total number of tested individuals. The seroprevalence studies were further classified into those carried out in symptomatic patients to diagnose acute infections and the ones carried out in asymptomatic individuals to measure past exposure or infection.

### Categorisation of infections

Infections were categorised as bacterial, fungal, parasitic, or viral and were sub-categorised using an epidemiological definition based on the predominant mode of transmission for the pathogens into mutually exclusive groups as contact (direct, indirect, droplet, or droplet nuclei transmission), vector-borne, airborne, and food- and/or water-borne [20].

### Study population and geographical classification

Study populations were grouped into four mutually exclusive categories: neonates (aged < 28 days), infants (1 to < 12 months), children (1 to < 13 years), and older individuals (≥ 13 years). If a study reported any participants from each age group, then they were grouped as participants of “all ages”. Countries were classified into sub-regions (“Southern Asia” and “South-eastern Asia”) according to United Nations designation of areas and regions [21].

### Statistical analyses and risk of bias assessment

The unit of analysis was a published article for each pathogen. Articles reporting a given pathogen were categorised by the UN sub-regions, patient age group of tested individuals, pathogen class (bacteria, viruses, fungi, and parasites), and predominant epidemiologic mode of transmission. The heterogeneity of study design, pathogens sought, laboratory methods, reporting, and limitations in data extraction precluded meta-analysis or estimation of pathogen prevalence. Multiple articles reporting different pathogens from the same study were treated as two separate articles. All analyses were carried out using R software version 3.6.0 (R Foundation for Statistical Computing, Vienna, Austria), and graphical presentations were done using ggplot2 library [22].

The currently available tools for assessing the quality and risk of bias were not applicable to our review design [23, 24]. We developed criteria specifically for quality assessment of the studies included in this review based on available information regarding study design and laboratory methods used for identification of the pathogens. We considered case reports and case series at a high risk of bias as they usually report atypical presentations. Seroprevalence studies were considered to be at moderate risk of bias as the distinction of acute and past infections depends on sample timing. For fever series, studies using culture, polymerase chain reaction (PCR) methods, or microscopy for detection of parasites were considered to be at low risk of bias. To assess whether published data were biased toward urban areas, the distance between the study location and the nearest major city was calculated using the coordinates of cities in SA and SEA available in the “maps” library [25].

## Results

The database search identified a total of 29,558 articles, with 20,442 and 9116 articles for SA and SEA, respectively. Of these, 2410 unique articles were selected for the final synthesis (Fig. 1). Among these, 1181 (49.0%) were case series, 585 (24.3%) were seroprevalence studies, 512 (21.2%) were fever series, and 132 (5.5%) articles comprised combinations of the aforementioned study types. Of 711 articles describing seroprevalence studies, 468 were in symptomatic patients, 67 were in asymptomatic participants, and 174 comprised of both groups (133 were conducted during outbreaks).

### Spatial distribution

**Fig. 2:**
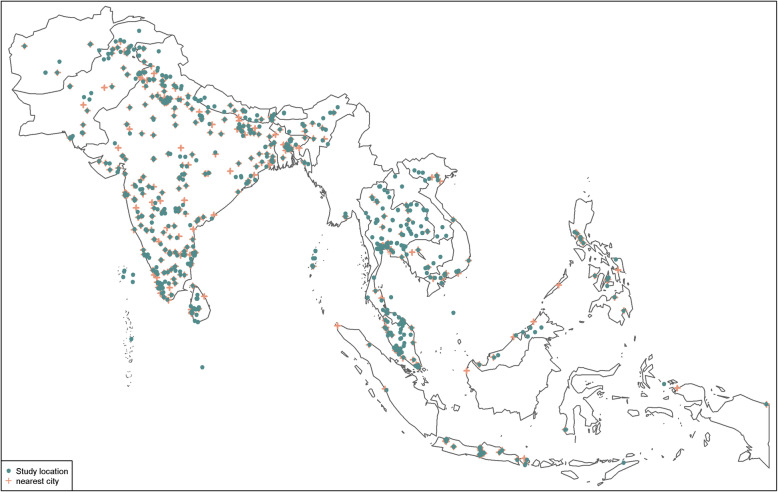
Location of study sites, systematic review of publications from Southern Asia and South-eastern Asia, 1980–2015. Location of study sites reported on in this review (in green) augmented with major cities (in red). Data on major cities were obtained from “maps” package in R software, and for the purpose of this review, only cities with population greater than 100,000 are shown

**Fig. 3:**
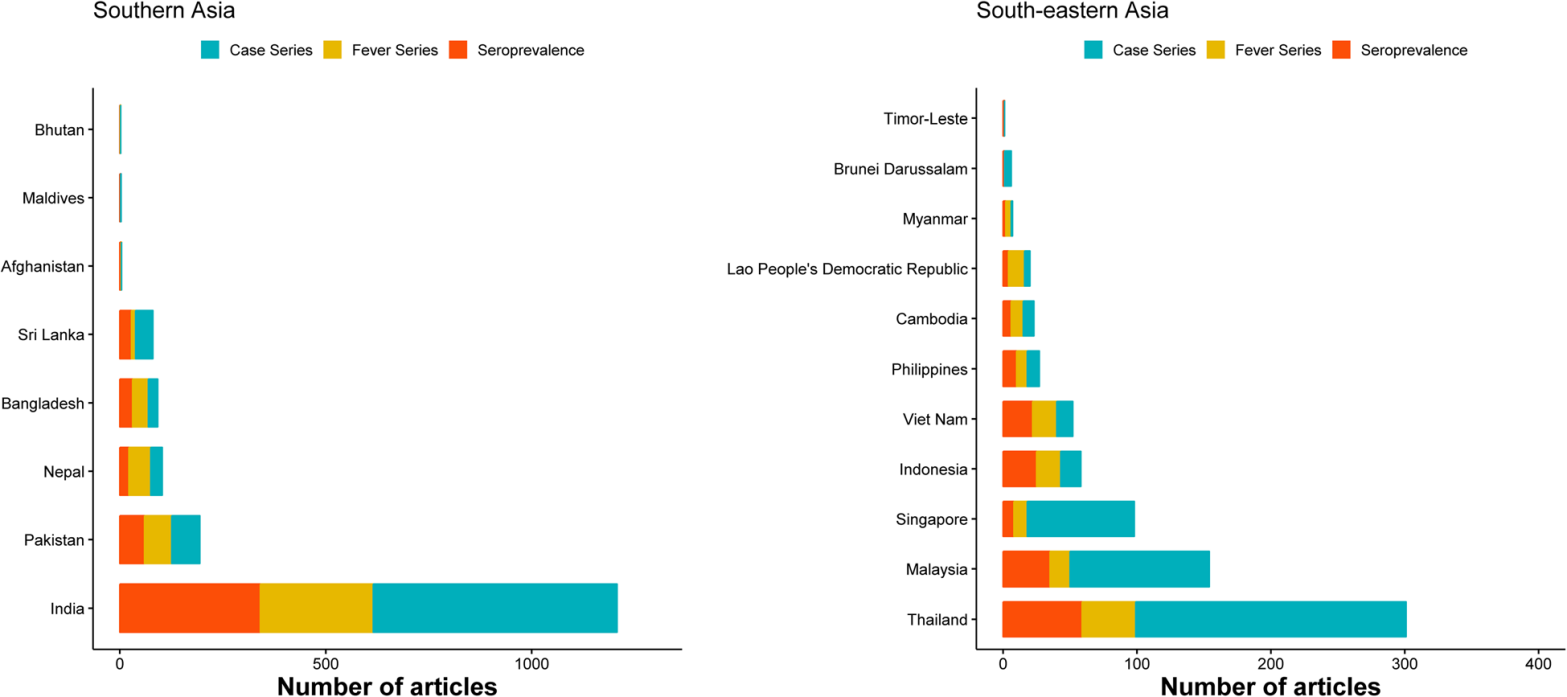
Number of publications by country, from Southern Asia and South-eastern Asia, 1980–2015. The total number of studies reported from each of the country over the review period from 1980 through 2015. Case series included individual case reports or series of patients with the same condition. Studies were classed as fever series if the total population denominator tested was reported and if an accurate diagnostic test for pathogen identification (culture or molecular methods) was used. Seroprevalence studies were defined as studies that reported the number of individuals testing positive using a serological test along with the total number of tested individuals

Data were collected from 2075 unique study sites, 1880 (90.6%) of which were within a radius of 50 km of the nearest city (Fig. 2). There were 1675 (69.5%) unique articles from SA, 732 (30.4%) from SEA, and three (0.1%) reported from both regions (Fig. 3). In SA, most reports were from India (*n* = 1207) followed by Pakistan (*n* = 194), Nepal (*n* = 102), Bangladesh (*n* = 92), and Sri Lanka (*n* = 80). Most reports from SEA were from Thailand (*n* = 301), followed by Malaysia (*n* = 154), Singapore (*n* = 98), Indonesia (*n* = 58), and Vietnam (*n* = 52). There were fewer than 10 reports from each of Afghanistan, Bhutan, Brunei Darussalam, Maldives, Myanmar, and Timor-Leste.

### Study population

**Fig. 4:**
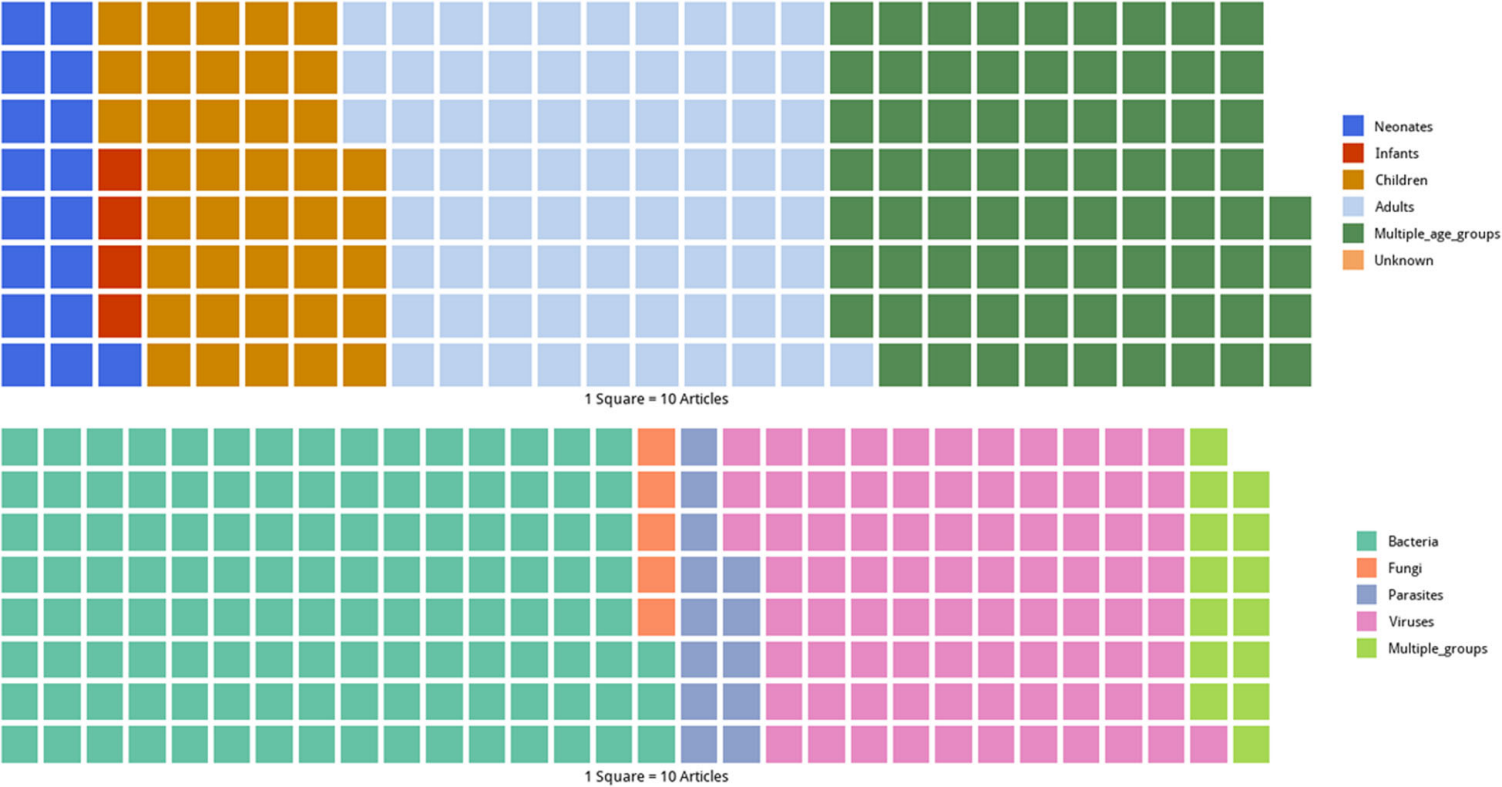
Waffle plots showing the distribution of articles by age categories (top) and pathogen categories (bottom), systematic review of published aetiological studies and case reports from Southern Asia and South-eastern Asia, 1980–2015

Studies reporting on neonates were found in 176 (7.3%) articles, infants in 47 (2.0%), children aged 1 to < 13 years in 408 (16.9%), and older children and adults (≥ 13 years) in 764 (31.7%). There were 757 (31.4%) articles that included participants of all ages, while the age group studied was not reported in 258 (10.7%) articles (Fig. 4; upper panel).

### Samples collected and diagnostic methods

Blood was the main specimen analysed in 2068 (85.8%) reports included in this review. Cerebrospinal fluid (CSF) samples were analysed in 101 (4.2%) articles; both CSF and blood in 60 (2.5%); bone marrow, joint, lymph, or liver aspirates in 63 (2.6%); peritoneal, pericardial, or pleural fluids in 23 (1.0%); spleen samples in 10 (0.4%); and vitreous humour samples in 10 (0.4%). Multiple sample sources were analysed in 73 (3.0%) articles, and the specimen analysed was not specified in two (0.1%) (Supplemental file 1; section 1.3).

Bacterial infections were detected using culture methods in 920 (66.8%) articles, serological assays in 340 (24.7%), PCR in 30 (2.2%), and microscopy and staining in seven (0.5%), and multiple diagnostic methods were reported in 89 (5.8%) articles. For viruses, 680 (75.5%) articles reported serological testing, and 90 (10.0%) PCR methods, 14 (1.6%) culture methods, and multiple diagnostic methods were reported in 117 (13.0%) articles. Fungal infections were identified using culture methods in 125 (87.4%) articles while parasites were detected using microscopy methods in 70 (51.1%) articles and serological tests in 39 (5.6%) articles. Further details are presented in Supplemental file 1; section 1.3.

### Aetiological findings

Bacterial infections were reported in 1235 (51.2%) articles, viral infections in 846 (35.1%), parasitic infections in 132 (5.5%), and fungal infections in 54 (2.2%), and 143 (6.0%) articles reported more than one pathogen group (Fig. 4; lower panel). The median number of pathogen species reported in a study was one [range 1–46, interquartile range 1–2]. Among 2410 articles, 1774 (73.6%) reported one species of microorganisms while 2 to < 5 microorganisms were reported in 319 (13.2%) articles, between 5 to < 10 in 192 (8.0%) articles, and ≥ 10 microorganisms in 125 (5.2%) articles.

### Bacterial infections

**Fig. 5:**
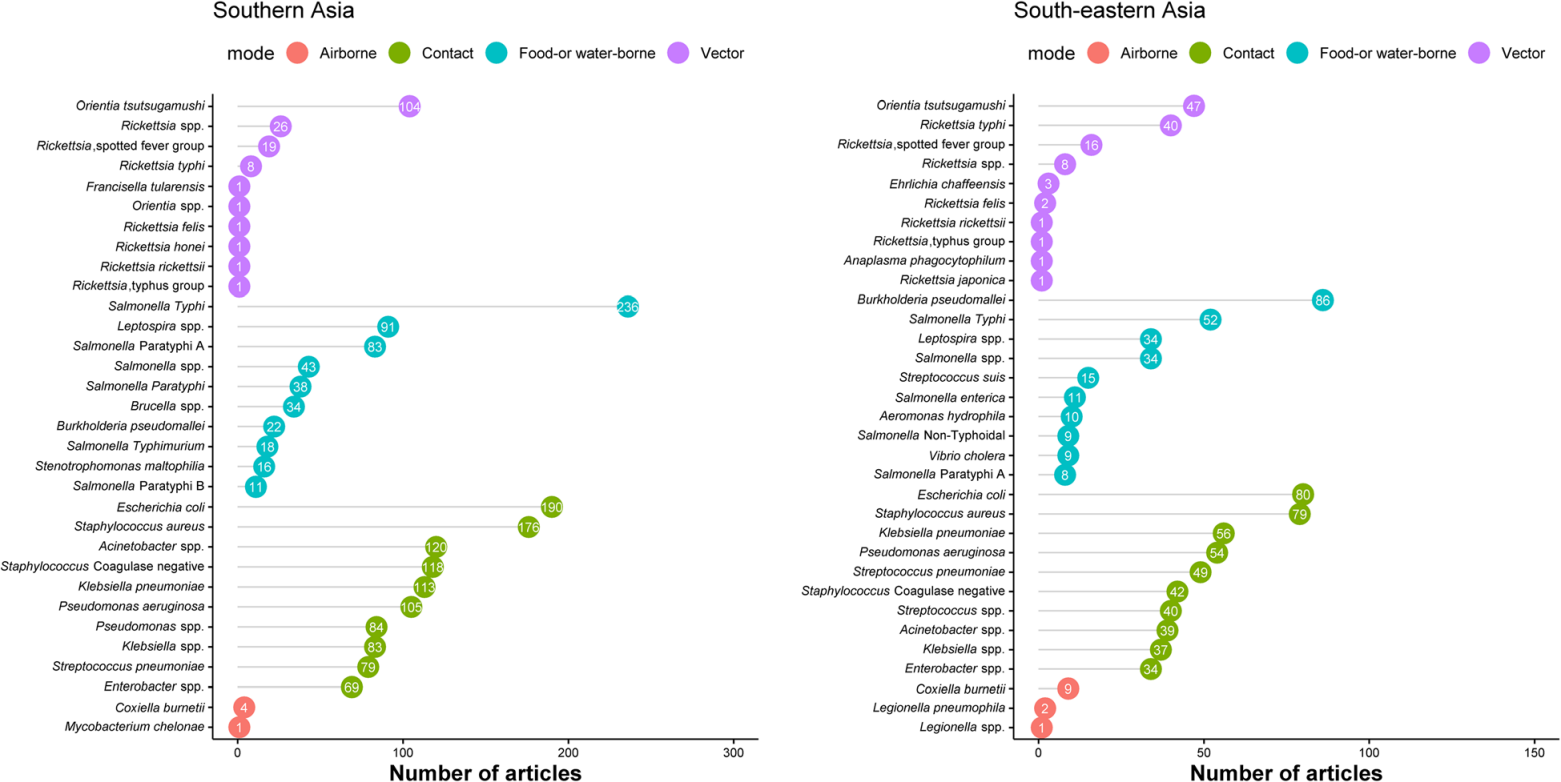
Most commonly reported bacterial infections by predominant mode of transmission, Southern and South-eastern Asia, 1980–2015. The graph presents the top 10 pathogens (based on the number of the published articles) by epidemiological mode of transmission

*Salmonella* Typhi (*n* = 285 articles), *Escherichia coli* (*n* = 270), *Staphylococcus aureus* (*n* = 255), *Klebsiella pneumoniae* (*n* = 169), and coagulase negative *Staphylococci* (*n* = 160) were the top five commonly reported bacteria (Fig. 5). *Salmonella* Typhi (*n* = 236) was the most commonly reported bacterium in Southern Asia (Fig. 5; left panel), whereas in South-eastern Asia, *Burkholderia pseudomallei* (*n* = 86) was the most commonly reported (Fig. 5; right panel).

#### Vector-borne bacterial infections

*Orientia tsutsugamushi* was the most commonly reported vector-borne bacterium (*n* = 151 articles) followed by *Rickettsia typhi* (*n* = 48); *Rickettsia*, spotted fever group (*n* = 37); and *Rickettsia* spp. (*n* = 34) (Fig. 5). *Orientia tsutsugamushi* was reported in 104 (6.2%) articles from Southern Asia and 47 (6.4%) from South-eastern Asia. There was a single report of *Borrelia burgdorferi* from India in 2010.

#### Food- and/or water-borne bacterial infections

*Salmonella* Typhi (*n* = 285 articles) was the most common cause of food- and/or water-borne bacterial infections, followed by *Leptospira* spp. (*n* = 125) and *Burkholderia pseudomallei* (*n* = 108). Among neonates, *Salmonella* spp. were reported in seven articles followed by *Burkholderia pseudomallei* (*n* = 6) and *Listeria monocytogenes* (*n* = 4); this was similar among children aged 1 to < 13 years. Among adolescent children and adults (≥ 13 years), *Burkholderia pseudomallei* (*n* = 58), *Leptospira* spp. (*n* = 44), and *Salmonella* Typhi (*n* = 37) were the most common reported pathogens. *Burkholderia pseudomallei* was reported in 86 articles from SEA and 22 from SA. *Streptococcus suis* was reported in SEA from Cambodia (*n* = 1; 2013), Lao PDR (*n* = 2; 2013–2015), Vietnam (*n* = 1; 2008), and Thailand (*n* = 11; 2003–2015).

#### Bacterial infections that spread through contact

*Escherichia coli* (*n* = 270 articles), *Staphylococcus aureus* (*n* = 255), and *Klebsiella pneumoniae* (*n* = 169) were the most commonly identified bacteria which spread through contact. Among neonates, *Klebsiella pneumoniae* was reported in 60 articles, while among children aged 1 to < 13 years, *Streptococcus pneumoniae* (*n* = 54), *Staphylococcus aureus* (*n* = 50), *Escherichia coli* (*n* = 45), *Haemophilus influenzae* (*n* = 31), and *Pseudomonas aeruginosa* (*n* = 27) were the top five commonly reported bacteria (Fig. 5).

#### Airborne bacterial infections

*Coxiella burnetii* was reported in 13 articles and *Legionella pneumophila* in two articles, and *Mycobacterium chelonae* and *Legionella* spp. were reported in an article each. Among neonates, *Coxiella burnetii* (*n* = 1) was the only description of airborne bacterial infection, while there were no reports among older children.

### Viral infections

**Fig. 6:**
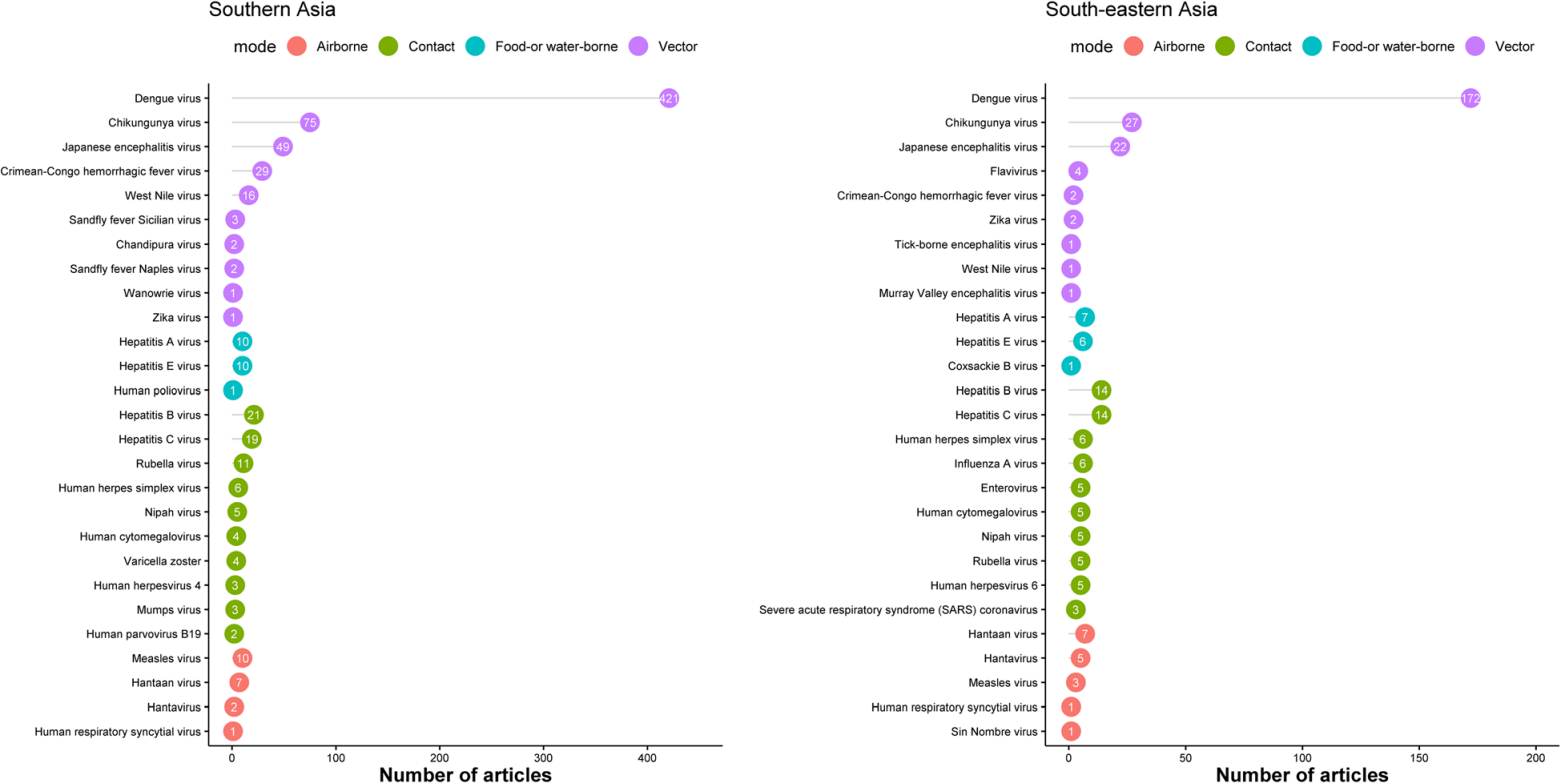
Most commonly reported viral infections by predominant mode of transmission, Southern and South-eastern Asia, 1980–2015. The graph presents the top 10 pathogens (based on the number of the published articles) by epidemiological mode of transmission

Dengue virus infection was the most commonly reported viral infection (*n* = 593) followed by chikungunya virus (*n* = 102), Japanese encephalitis virus (*n* = 71), hepatitis B virus (*n* = 35), and hepatitis C virus (*n* = 33) (Fig. 6). Among neonates, the distribution of viral infections differs with 13 articles reporting dengue virus infections (*n* = 13), herpes simplex virus (*n* = 3), chikungunya virus (*n* = 2), enterovirus (*n* = 2), Japanese encephalitis virus (*n* = 1), and flavivirus (*n* = 1). Among infants, dengue virus (*n* = 8), rubella virus (*n* = 2), and an article each reported: chikungunya virus, Coxsackie B virus, hepatitis B virus, human herpesvirus 6, and B19 virus. Among children, dengue virus (*n* = 118), Japanese encephalitis virus (*n* = 28), measles virus (*n* = 9), hepatitis A virus (*n* = 7), and human herpes simplex virus (*n* = 7) were the top five commonly reported viruses. Among adults, dengue virus (*n* = 184), chikungunya virus (*n* = 28), Crimean-Congo haemorrhagic fever virus (*n* = 16), hepatitis B virus (*n* = 15), and Japanese encephalitis virus (*n* = 12) were the top five reported viruses. The age-stratified distribution of the reports on viral infections is presented in Supplemental file 1; section 1.3.

#### Vector-borne viruses

Dengue virus was the most commonly reported arbovirus (*n* = 593) followed by chikungunya virus (*n* = 102), Japanese encephalitis virus (*n* = 71), Crimean-Congo haemorrhagic fever virus (*n* = 31), and West Nile virus (*n* = 17). Zika virus was reported in an article each from Indonesia (1981), Pakistan (1983), and the Philippines (2015).

#### Food- and/or water-borne viral infections

Hepatitis A virus was reported in 17 articles and hepatitis E virus in 16. Human poliovirus was reported in an article from India (1991), and there was one report describing Coxsackie B virus from Thailand (1991). There were no other descriptions of food- and/or water-borne viral infections.

#### Airborne viral infections

Hantaan virus (*n* = 14), measles virus (*n* = 13), hantavirus (*n* = 7), human respiratory syncytial virus (*n* = 2 articles), and Sin Nombre virus (*n* = 1) were the airborne viral infections reported. Hantavirus was reported in an article each from Cambodia, Indonesia, and Thailand and in two articles each from India and Singapore.

#### Viral infections spreading through contact

Hepatitis B virus (*n* = 35), hepatitis C virus (*n* = 33), rubella virus (*n* = 16), and human herpes simplex virus (*n* = 12) were the most commonly reported viruses spreading through contact. Nipah virus was reported from Malaysia (*n* = 3, 1999–2002), Singapore (*n* = 2, 1999–2001), India (*n* = 1, 2006), and Bangladesh (*n* = 4, 2006–2012).

### Parasitic and fungal infections

**Fig. 7:**
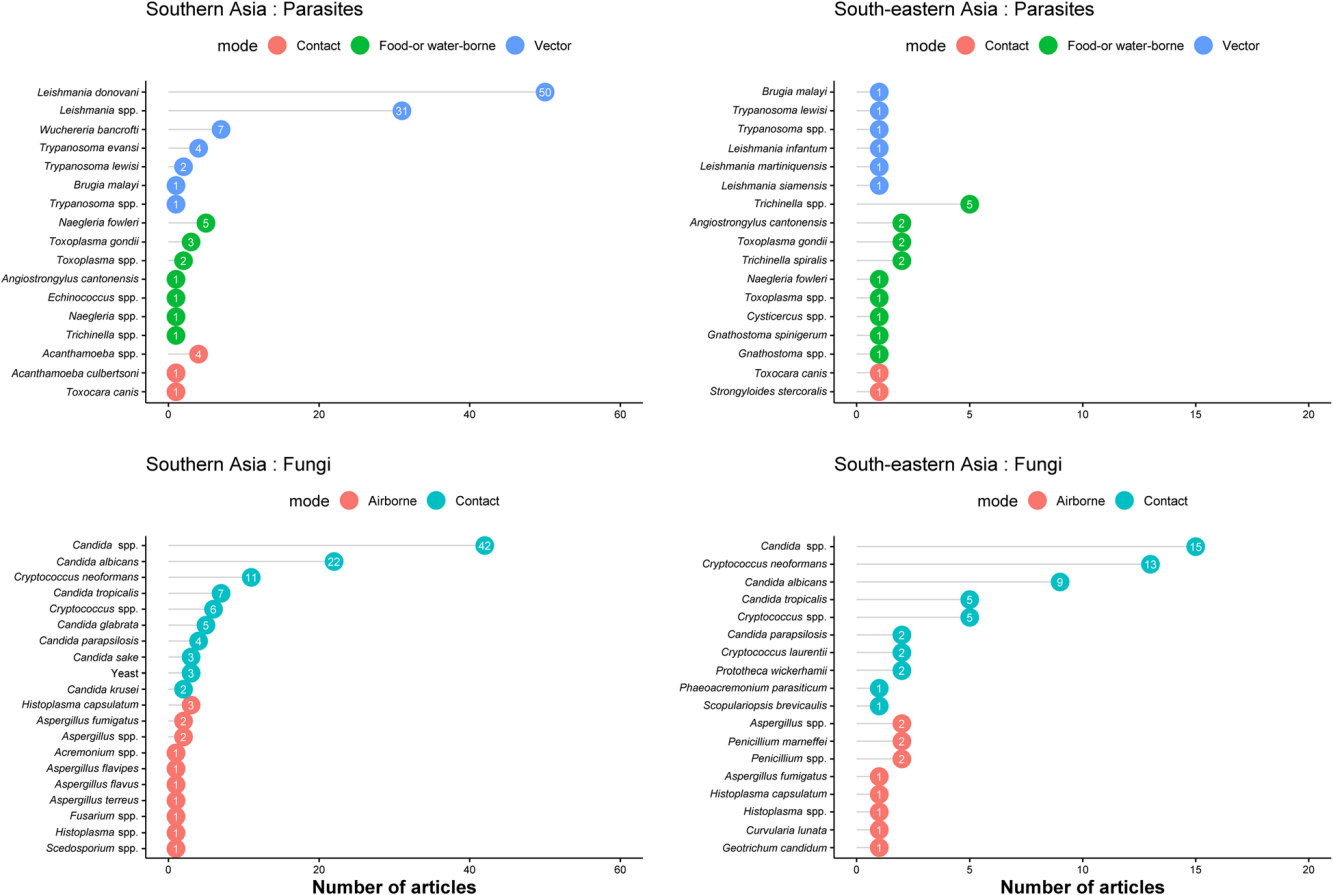
All reported parasitic and fungal infections by predominant mode of transmission, Southern and South-eastern Asia, 1980–2015. The graph presents the top 10 pathogens (based on the number of the published articles) by epidemiological mode of transmission

*Leishmania donovani* was the most reported parasite (*n* = 50 articles) followed by *Leishmania* spp. (*n* = 31), *Wuchereria bancrofti* (*n* = 7), *Naegleria fowleri* (*n* = 6), and *Trichinella* spp. (*n* = 6). All of the reported *Leishmania donovani* were from Southern Asia (Fig. 7). Among fungi, *Candida* spp. (*n* = 90) were the most commonly reported, with 31 articles describing *Candida albicans*, followed by *Cryptococcus neoformans* (*n* = 24) and *Candida tropicalis* (*n* = 12) (Fig. 7). The majority of the reported *C. neoformans* were in HIV-uninfected patients.

### Spatial and temporal trends in infectious causes of fever

The temporal trend in most commonly reported fever-causing pathogens over the different time periods is presented in Tables 1, 2, and 3. In SA, the leading reported bacterial cause of NMFI was *Salmonella* Typhi whereas *Burkholderia pseudomallei* was the most commonly reported cause of NMFI in SEA (Fig. 5). Dengue virus was the most commonly reported viral cause across different time periods in both SA and SEA (Fig. 6). Among parasites, *Leishmania* spp. were the most commonly reported parasites in SA, but were not commonly reported in SEA (Fig. 7). Literature on fungi mostly reported *Candida* spp. and *Cryptococcus* spp. (mostly from HIV-uninfected patients) across the time periods and regions (Fig. 7).

**Table 1:** Top five most commonly reported pathogens in Southern Asia, stratified by time period

	1980 to ≤ 1990	1991 to ≤ 2000	2001 to ≤ 2010	2011 to ≤ 2015
**Bacteria**	*Salmonella* Typhi (*n* = 12)	*Salmonella* Typhi (*n* = 51)	*Salmonella* Typhi (*n* = 96)	*Escherichia coli* (*n* = 111)
	*Salmonella* Paratyphi A (*n* = 5)	*Staphylococcus aureus* (*n* = 16)	*Escherichia coli* (*n* = 63)	*Staphylococcus aureus* (*n* = 98)
	*Salmonella* spp. (*n* = 2)	Coagulase negative *Staphylococcus* (*n* = 14)	*Staphylococcus aureus* (*n* = 61)	*Salmonella* Typhi (*n* = 77)
	*Pseudomonas aeruginosa* (*n* = 2)	*Escherichia coli* (*n* = 14)	*Leptospira* spp. (*n* = 46)	*Orientia tsutsugamushi* (*n* = 74)
	*Klebsiella* spp. (*n* = 2)	*Klebsiella pneumoniae* (*n* = 13)	*Streptococcus pneumoniae* (*n* = 38)	*Acinetobacter* spp. (*n* = 72)
**Viruses**	Japanese encephalitis virus (*n* = 5)	Dengue virus (*n* = 41)	Dengue virus (*n* = 166)	Dengue virus (*n* = 210)
	Dengue virus (*n* = 4)	Japanese encephalitis virus (*n* = 5)	Chikungunya virus (*n* = 36)	Chikungunya virus (*n* = 38)
	West Nile virus (*n* = 3)	Hepatitis B virus (*n* = 4)	Japanese encephalitis virus (*n* = 16)	Japanese encephalitis virus (*n* = 23)
	CCHF virus (*n* = 3)	West Nile virus (*n* = 3)	CCHF virus (*n* = 11)	CCHF virus (*n* = 13)
	Sandfly fever Sicilian virus (*n* = 2)	Hepatitis C virus (*n* = 3)	Hepatitis C virus (*n* = 8)	West Nile virus (*n* = 8)
**Parasites**	*Leishmania donovani* (*n* = 3)	*Leishmania* spp. (*n* = 12)	*Leishmania donovani* (*n* = 25)	*Leishmania* spp. (*n* = 13)
	*Toxoplasma* spp. (*n* = 1)	*Leishmania donovani* (*n* = 10)	*Leishmania* spp. (*n* = 6)	*Leishmania donovani* (*n* = 12)
	*Toxocara canis* (*n* = 1)	*Wuchereria bancrofti* (*n* = 3)	*Naegleria fowleri* (*n* = 4)	*Trypanosoma lewisi* (*n* = 2)
	*Acanthamoeba culbertsoni* (*n* = 1)	*Naegleria* spp. (*n* = 1)	*Wuchereria bancrofti* (*n* = 3)	*Wuchereria bancrofti* (*n* = 1)
	–	*Brugia malayi* (*n* = 1)	*Trypanosoma evansi* (*n* = 3)	*Trypanosoma evansi* (*n* = 1)
**Fungi**	*Candida* spp. (*n* = 1)	*Candida albicans* (*n* = 5)	*Candida* spp. (*n* = 10)	*Candida* spp. (*n* = 29)
	–	*Candida* spp. (*n* = 2)	*Cryptococcus neoformans* (*n* = 7)	*Candida albicans* (*n* = 11)
	–	*Cryptococcus* spp. (*n* = 1)	*Candida albicans* (*n* = 6)	*Candida tropicalis* (*n* = 4)
	–	*Cryptococcus neoformans* (*n* = 1)	Yeast (*n* = 3)	*Candida glabrata* (*n* = 4)
	–	*Candida tropicalis* (*n* = 1)	*Cryptococcus* spp. (*n* = 3)	*Cryptococcus neoformans* (*n* = 3)

*CCHF* Crimean-Congo haemorrhagic fever virus. The number in parentheses indicates the number of publications reporting the given microorganism

**Table 2:** Top five most commonly reported pathogens in South-eastern Asia, stratified by time period

	1980 to ≤ 1990	1991 to ≤ 2000	2001 to ≤ 2010	2011 to ≤ 2015
**Bacteria**	*Burkholderia pseudomallei* (*n* = 11)	*Burkholderia pseudomallei* (*n* = 16)	*Burkholderia pseudomallei* (*n* = 34)	*Staphylococcus aureus* (*n* = 26)
	*Staphylococcus aureus* (*n* = 9)	*Staphylococcus aureus* (*n* = 15)	*Escherichia coli* (*n* = 32)	*Escherichia coli* (*n* = 26)
	*Escherichia coli* (*n* = 8)	*Escherichia coli* (*n* = 14)	*Staphylococcus aureus* (*n* = 29)	*Burkholderia pseudomallei* (*n* = 25)
	Coagulase negative *Staphylococcus* (*n* = 7)	*Klebsiella pneumoniae* (*n* = 13)	*Salmonella* Typhi (*n* = 25)	*Klebsiella pneumoniae* (*n* = 18)
	*Orientia tsutsugamushi* (*n* = 7)	*Streptococcus pneumoniae* (*n* = 10)	*Pseudomonas aeruginosa* (*n* = 23)	*Streptococcus pneumoniae* (*n* = 16)
**Viruses**	Dengue virus (*n* = 13)	Dengue virus (*n* = 31)	Dengue virus (*n* = 86)	Dengue virus (*n* = 42)
	Japanese encephalitis virus (*n* = 3)	Japanese encephalitis virus (*n* = 5)	Chikungunya virus (*n* = 14)	Chikungunya virus (*n* = 13)
	Human cytomegalovirus (*n* = 2)	Nipah virus (*n* = 3)	Japanese encephalitis virus (*n* = 10)	Hepatitis B virus (*n* = 5)
	Hantavirus (*n* = 2)	Human herpesvirus 6 (*n* = 3)	Hepatitis C virus (*n* = 10)	Japanese encephalitis virus (*n* = 4)
	Hantaan virus (*n* = 2)	Hepatitis C virus (*n* = 2)	Hepatitis B virus (*n* = 8)	Human herpes simplex virus (*n* = 4)
**Parasites**	*Naegleria fowleri* (*n* = 1)	*Trichinella* spp. (*n* = 2)	*Trichinella spiralis* (*n* = 2)	*Trichinella* spp. (*n* = 3)
	–	*Toxoplasma gondii* (*n* = 1)	*Angiostrongylus cantonensis* (*n* = 2)	*Toxoplasma gondii* (*n* = 1)
	–	*Toxocara canis* (*n* = 1)	*Trypanosoma* spp. (*n* = 1)	*Leishmania siamensis* (*n* = 1)
	–	*Cysticercus* spp. (*n* = 1)	*Trypanosoma lewisi* (*n* = 1)	*Leishmania martiniquensis* (*n* = 1)
	–	–	*Toxoplasma* spp. (*n* = 1)	*Gnathostoma* spp. (*n* = 1)
**Fungi**	*Cryptococcus neoformans* (*n* = 2)	*Cryptococcus neoformans* (*n* = 3)	*Cryptococcus neoformans* (*n* = 6)	*Candida* spp. (*n* = 10)
	*Penicillium marneffei* (*n* = 1)	*Prototheca wickerhamii* (*n* = 1)	*Candida* spp. (*n* = 4)	*Candida albicans* (*n* = 6)
	*Cryptococcus laurentii* (*n* = 1)	*Histoplasma capsulatum* (*n* = 1)	*Candida tropicalis* (*n* = 2)	*Cryptococcus* spp. (*n* = 4)
	*Cryptococcus albidus* (*n* = 1)	*Hansenula anomala* (*n* = 1)	*Candida albicans* (*n* = 2)	*Penicillium* spp. (*n* = 2)
	*Candida* spp. (*n* = 1)	*Geotrichum candidum* (*n* = 1)	*Scopulariopsis brevicaulis* (*n* = 1)	*Cryptococcus neoformans* (*n* = 2)

The number in parentheses indicates the number of publications reporting the given microorganism

**Table 3:** Commonly reported bacterial pathogens among neonates, stratified by sub-regions and time period

	1980 to ≤ 1990	1991 to ≤ 2000	2001 to ≤ 2010	2011 to ≤ 2015
SA	*Citrobacter* spp. (*n* = 2)	Coagulase negative *Staphylococcus* (*n* = 6)	*Escherichia coli* (*n* = 26)	*Escherichia coli* (*n* = 44)
	*Streptococcus* spp. (*n* = 1)	*Staphylococcus aureus* (*n* = 6)	*Staphylococcus aureus* (*n* = 24)	*Staphylococcus aureus* (*n* = 39)
	Coagulase negative *Staphylococcus* (*n* = 1)	*Klebsiella pneumoniae* (*n* = 4)	*Klebsiella pneumoniae* (*n* = 19)	Coagulase negative *Staphylococcus* (*n* = 37)
	*Staphylococcus aureus* (*n* = 1)	*Escherichia coli* (*n* = 4)	*Acinetobacter* spp. (*n* = 15)	*Klebsiella pneumoniae* (*n* = 31)
	*Salmonella* spp. (*n* = 1)	*Acinetobacter* spp. (*n* = 4)	Coagulase negative *Staphylococcus* (*n* = 13)	*Acinetobacter* spp. (*n* = 28)
SEA	*Staphylococcus* aureus (*n* = 4)	*Streptococcus agalactiae* (*n* = 2)	*Klebsiella* spp. (*n* = 3)	*Escherichia coli* (*n* = 7)
	Coagulase negative *Staphylococcus* (*n* = 3)	*Streptococcus*, viridans group (*n* = 1)	*Streptococcus agalactiae* (*n* = 2)	*Staphylococcus aureus* (*n* = 6)
	*Escherichia coli* (*n* = 3)	Coagulase negative *Staphylococcus* (*n* = 1)	*Stenotrophomonas* spp. (*n* = 2)	*Streptococcus agalactiae* (*n* = 5)
	*Streptococcus* spp. (*n* = 2)	*Staphylococcus aureus* (*n* = 1)	Coagulase negative *Staphylococcus* (*n* = 2)	*Acinetobacter baumannii* (*n* = 5)
	*Streptococcus pyogenes* (*n* = 2)	*Pseudomonas* spp. (*n* = 1)	*Pseudomonas* spp. (*n* = 2)	Coagulase negative *Staphylococcus* (*n* = 4)

*SA* Southern Asia, *SEA* South-eastern Asia. The number in parentheses indicates the number of publications reporting the given microorganism

### Pathogens of regional interest or considered as emerging

**Fig. 8:**
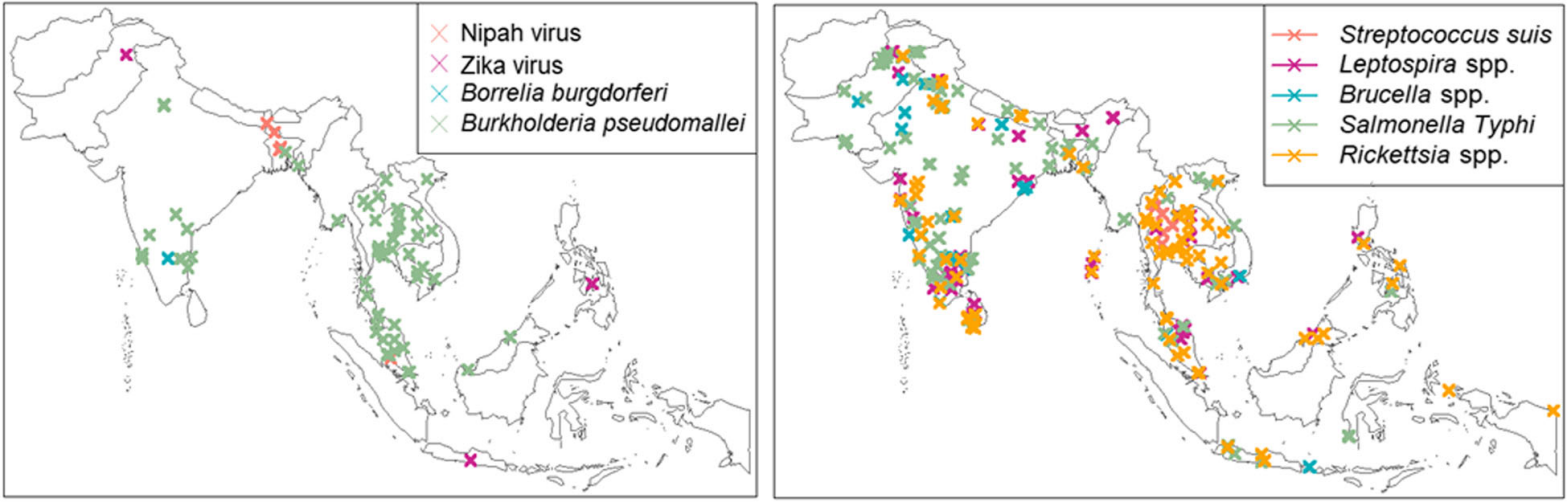
Pathogens of regional interest or emerging pathogens, publications from Southern Asia and South-eastern Asia, 1980–2015. The map shows the location of study sites reporting each pathogen

The distribution of the study sites for some of the pathogens (Nipah virus, *Borrelia burgdorferi*, Zika virus) considered as emerging or of regional interest is presented in Fig. 8. *Streptococcus suis* was reported only in SEA, from Cambodia (*n* = 1; 2013), Lao PDR (*n* = 2; 2013–2015), Vietnam (*n* = 1; 2008), and Thailand (*n* = 11; 2003–2015). Nipah virus was reported from Malaysia (*n* = 3; 1999–2002), Singapore (*n* = 2; 1999–2001), India (*n* = 1; 2006), and Bangladesh (*n* = 4; 2006–2012).

### Assessment of risk of bias

Of 2410 included articles, the risk of bias was considered to be moderate or high in 1181 (49.0%) and low in 1229 (51.0%). Of 2075 studies where it could be ascertained, 1880 (90.6%) were from urban settings or located within a radius of 50 km of the capital city or another major city (Supplemental file 2).

## Discussion

In order to characterise the aetiological agents of febrile illnesses in the region, we screened approximately 30,000 articles and present here the findings from 2410 articles published from 1980 through 2015, the largest systematic review to date on the topic.

The review yielded several findings relevant for the management of febrile illness. *Salmonella* Typhi was the most common cause of bacterial infections in Southern Asia whereas *Burkholderia pseudomallei*, the causative agent of melioidosis, was the most commonly reported bacterial infection in SEA. The latter was also reported in 22 articles from India and Bangladesh. A recent modelling study has suggested that melioidosis continues to be under-diagnosed and under-reported globally [26]. The high case fatality ratio and the under-diagnosis of the disease highlight the urgent need for raising awareness of its presence as a causative pathogen and improving access to diagnosis in Southern Asia. Similarly, vector-borne infections such as mite-borne *Orientia tsutsugamushi*, the causative pathogen of scrub typhus, were reported across the region but with notable geographical gaps with no reports identified from Myanmar, Pakistan, or Bhutan among others in the purported “tsutsugamushi triangle” [27].

Viral aetiologies were reported in 35% of the articles with the majority reporting dengue, chikungunya, or Japanese encephalitis virus infections. As the most reported viral infections, it is noteworthy that dengue and chikungunya viruses cause diseases with overlapping symptoms and hence are prone to misdiagnosis, as co-infections and co-distribution have been reported in SA and SEA [28]. Our study identified three reports of Zika virus (two in the 1980s and one in 2015). Although only sporadic cases have been reported from Asia, there is a potential for future outbreaks given the dense growing population, rapid urbanisation, and presence of the vector, among other factors [29].

Our review has shown a large heterogeneity in study site selection, design, conduct, and reporting on studies of infections that may cause fever. Most of the studies included in this review were carried out in major cities or within close distance from major cities (less than 50 km) or the national capital. The selection of study sites is often influenced by academic institutions, logistical considerations, and funding availability. This limits the generalizability of the results to the whole country or region and clearly represents a reporting bias toward urban settings, in these regions with a predominantly rural population, as has been reported earlier in a systematic review aimed at characterising febrile illness in the Greater Mekong sub-region [9]. Similarly, the age range of the study population tested was not reported in one out of every 10 articles, despite this being critical for interpretation of the epidemiological findings and for guiding preventive and therapeutic control recommendations. The incidence along with the associated morbidity and mortality of different aetiologies varies between different age groups. Furthermore, absence of data on age limits the usefulness of these datasets for further inclusion in meta-analyses. Similarly, reporting of laboratory methods was variable. The recently published Microbiology Investigation Criteria for Reporting Objectively (MICRO) guideline which aims to standardise reporting of such studies might be a useful resource to consider for reporting and reviewing aetiological studies [30].

Our review shares many of the limitations discussed in the preceding articles in this series [31]. As in the NMFI Africa review, restricting the inclusion criteria to studies investigating blood and other sterile fluids meant that several important causes of NMFI were excluded. Two examples of this in SA and SEA are enterovirus and influenza infections. A very low number of articles describing these were identified, despite the known high burden of hand, foot, and mouth disease and viral respiratory illnesses in the region [32]. These viruses are usually diagnosed by PCR analysis of the nasopharyngeal or throat swabs, or faecal samples, and hence, the stringent criteria of including only those articles that analysed sterile sample sources explain this discrepancy. Of note, the absence of reports on a specific pathogen from a country cannot be taken as definitive evidence of its absence. Our review found very few reports of a pathogen being tested for and found to be negative. Some of the reasons behind this could be publication bias through publication of reports with only positive findings, the diagnostic testing being driven by the interest of the researchers, or the availability/or lack of the relevant diagnostic resources.

There were some additional limitations specific to this review. We inadvertently failed to include studies from Iran, formally recognised as a part of Southern Asia by the UN. We also searched for articles only in a limited number of languages and did not search for publications in local languages. The articles were assessed by one independent reviewer owing to the broad scale of this review. Finally, a large proportion of the studies included in this review were considered to be at high risk of bias and the results of this systematic review should be interpreted taking this into consideration (Supplemental file 2).

Despite the limitations, our review provides several noteworthy observations that warrant additional awareness among researchers and clinicians in the region. Lyme disease, a tick-borne disease usually reported in Europe and North America [33], has seldom been reported in SA with only a few case reports found in the literature recently [34–36]. Praharaj et al. reported a prevalence of 13% (65/500) of anti-*Borrelia burgdorferi* antibodies in a seroprevalence study conducted in 2008 in North-Eastern states of India bordering with China [37]. Moreover, the report of Lyme disease from southern India (Karnataka state) in 2010 [38] along with other recent publications suggests that the disease might potentially pose an emerging threat in the Indian sub-continent and should be considered in the differential diagnosis of NMFIs. Nipah virus, a highly pathogenic paramyxovirus, now listed as a priority pathogen by the WHO, was reported in 10 articles from Malaysia, Singapore, India, and Bangladesh. Similarly, *Streptococcus suis* was reported in 15 articles from 2003 through 2015, all of which were in SEA countries. The high density of pig farming in the region and the relation to dietary preferences are thought to contribute to the transmission of this bacterium which is an important cause of meningitis in the region [39]. In regions with a high density of pig farming, this should be considered as a potential cause of febrile illnesses.

Some of these infections can be relatively benign and self-limiting (e.g. some viral infections) while others (e.g. *Burkholderia pseudomallei*, Nipah virus) without omitting malaria are life threatening. Southern Asia and South-eastern Asia are major hotspots for infectious diseases with almost a third of humanity being at risk [12]. A non-localised febrile illness is a common feature of these infectious diseases at presentation. Accurate and prompt diagnosis can be difficult in the absence of reliable point-of-care diagnostics, resulting in misdiagnosis and inappropriate prescription of broad-spectrum antimicrobials [40–42]. This highlights an urgent need for development of epidemiologically targeted diagnostic tools for detecting the causes of febrile illness.

## Conclusions

The map of pathogen distributions presented in this review provides regional data that can form a basis for targeted development of diagnostic tools and fever case management strategies in SA and SAE.

## Supplementary information

**Additional file 1.**

**Additional file 2.**

## Data Availability

All data generated and analysed in this review are openly available from the IDDO webpage as a downloadable resource (https://www.iddo.org/non-malarial-febrile-illness-home).

## Abbreviations

AMR: Antimicrobial resistance
BDSP: Banque de Données Santé Publique
CSF: Cerebrospinal fluid
DOI: Digital object identifier
MeSH: Medical Subject Headings
MICRO: Microbiology Investigation Criteria for Reporting Objectively
NMFI: Non-malarial febrile illness
PCR: Polymerase chain reaction
PRISMA: Preferred Reporting Items for Systematic Reviews and Meta-Analyses
RDT: Rapid diagnostic test
SA: Southern Asia
SEA: South-eastern Asia
URL: Uniform Resource Locator
WHO: World Health Organization
