# Supplementary material for: Non-malarial febrile illness: a systematic review of published aetiological studies and case reports from Southern Asia and South-eastern Asia, 1980–2015
Source: BMC Med. 2020 Sep 21;18:299. doi: 10.1186/s12916-020-01745-0 (PMC7504862; doi:10.1186/s12916-020-01745-0)
Supplement: Supplementary file 1 — Additional file 1. [file 12916_2020_1745_MOESM1_ESM.docx]

**Supplemental file 1:**

**Non-malarial febrile illness: A systematic review of published aetiological studies and case reports from Southern Asia and South- eastern Asia, 1980-2015**

Poojan Shrestha^1,2*^, Prabin Dahal^1,2^, Chinwe Ogbonnaa-Njoku^1,2^, Debashish Das^1,2^, Kasia Stepniewska^1,2^, Nigel V Thomas^1,2^, Heidi Hopkins^3^, John A Crump^4^, David Bell^6^, Paul N Newton^1,2,3,5^, Elizabeth A Ashley^2,5^, Philippe J Guérin^1,2*^

^1^ Infectious Diseases Data Observatory (IDDO), Oxford, UK

^2^ Centre for Tropical Medicine and Global Health, Nuffield Department of Clinical Medicine,

University of Oxford, Oxford, UK

^3^ London School of Hygiene and Tropical Medicine, London, UK

^4^ Centre for International Health, University of Otago, New Zealand

^5^ Lao-Oxford-Mahosot Hospital-Wellcome Trust Research Unit, Vientiane, Laos

^6^ Independent consultant, Issaquah, Washington, USA

^*^Correspondence to:

Dr. Poojan Shrestha

Email: [poojan.shrestha@iddo.org](mailto:poojan.shrestha@iddo.org)

Infectious Diseases Data Observatory, University of Oxford,

NDMRB, Old Road Campus, Roosevelt Drive, Headington, Oxford, OX3 7FZ, UK

*and*

Prof Philippe Guérin

Email: [philippe.guerin@iddo.org](mailto:philippe.guerin@iddo.org)

Infectious Diseases Data Observatory, University of Oxford,

NDMRB, Old Road Campus, Roosevelt Drive, Headington, Oxford, OX3 7FZ, UK

### **Supplemental file 1: Further details on the systematic review and data extraction**

### **1.1 Search terminologies used and the time-period used for searching different**

### **database**

For South Asia, the electronic database search was carried out as follows: EMBASE from 1980 through 2015, with the date of last search 4th August 2015, MEDLINE between 1946 to 2015 with last search done on 13^th^ July 2015. Global Health between 1910 to 2015 with last search date 4^th^ August 2015. The WHO Global Health Library (SEARO & WPRO files) was searched on 16^th^ July 2015.

For South-East Asia, searches were carried out in Global Health between 1910 and 2016, with last search done on 18^th^ April 2016. EMBASE was searched between 1980 to 2016 with last search done on 18^th^ April 2016. WHO Global Health Library (WPRO & SEARO Files) was searched on 19^th^ April 2016. BDSP was searched between 1980 to 2015, with last search on 31^st^ May 2016. PASCAL was searched between 1980 to 2015, and the last search was carried out on 2^nd^ August 2016.

**Example of a search string used for the MEDLINE database.**

**South Asia - Ovid MEDLINE(R) In-Process & Other Non-Indexed Citations, Ovid MEDLINE(R) Daily, Ovid MEDLINE(R) and Ovid OLDMEDLINE(R) <1946 to Present> - Searched 13th July 2015**

1 exp Anti-Bacterial Agents/dt, pd, tu, th, ut [Drug Therapy, Pharmacology, Therapeutic Use, Therapy, Utilization] (396988)

2 exp Bacteremia/ or (bacteremi* or bacteraemi* or septicemi* or septicaemi*).ti,ab. (42134)

3 exp Sepsis/ or ("blood stream infection*" or "bloodstream infection*" or "blood stream pathogen*" or "bloodstream pathogen*" or (("blood borne" or bloodborne or blood-borne) adj (infection* or pathogen*))).ti,ab. (103158)

4 exp Fever/ or (febrile or fever* or pyrexia or pyrexic or pyrexial or pyrexiae or hyperthermi* or (raised adj2 temperature) or (high adj2 temperature) or (elevated adj2 temperature)).ti,ab. (194090)

5 or/1-3 (501691)

6 (babesi* or leishmania* or chagas or trypanosom* or "sleeping sickness").ti,ab. (61688)

7 Babesia microti/ (225)

8 exp Trypanosoma/ (21833)

9 exp Trypanosomiasis/ not Trypanosomiasis, Bovine/ (17824)

10 exp Leishmania/ (16157)

11 exp Leishmaniasis/ (18280)

12 Influenza A Virus, H1N1 Subtype/ or Influenza A virus/ or Influenza a virus.ti,ab. (31204)

13 exp Arbovirus infections/ or Arboviruses/ or exp Encephalitis viruses, Japanese/ or Encephalitis virus, Japanese/ or Encephalitis virus, Murray Valley/ or "Encephalitis virus, St. Louis"/ or West Nile virus/ or Encephalitis viruses, tick-borne/ or Yellow fever virus/ or (Japanese Encephalitis or Japanese B Encephalitis or Phlebotomus fever or Rift Valley fever or Yellow fever or ((California or "St Louis" or Tick-borne) adj Encephalitis) or Kyasanur Forest disease).ti,ab. (37910)

14 Chikungunya virus/ or Chikungunya.ti,ab. (2362)

15 exp Epstein-Barr Virus Infections/ or Epstein-Barr.ti,ab. (41647)

16 exp Dengue/ or Dengue Virus/ or Dengue.ti,ab. (14155)

17 Respiratory Syncytial Virus, Human/ or Respiratory Syncytial Virus Infections/ or (Respiratory Syncytial Virus or RSV).ti,ab. (13604)

18 Measles/ or Measles virus/ or Measles.ti,ab. (23006)

19 Hantavirus/ or Hantaan Virus/ or Puumala Virus/ or Seoul Virus/ or Sin Nombre Virus/ or Hantavirus Infections/ or Hantavirus Pulmonary Syndrome/ or Hemorrhagic Fever with Renal Syndrome/ or (Hantavirus or Hantaan or Puumula or Seoul virus or Sin Nombre virus or Hemorrhagic fever or Haemorrhagic fever).ti,ab. (10240)

20 Nipah Virus/ or Nipah Virus.ti,ab. (592)

21 Coxsackievirus Infections/ or (Coxsackie or Coxsackievirus*).ti,ab. (8652)

22 Cytomegalovirus Infections/ or Cytomegalovirus/ or Cytomegalovirus*.ti,ab. (42714)

23 Hepatitis/ or Hepatitis, Viral, human/ or Hepatitis a/ or Hepatitis b/ or Hepatitis b, chronic/ or Hepatitis c/ or Hepatitis c, chronic/ or Hepatitis e/ or Hepatitis b virus/ or Hepacivirus/ or Hepatitis e virus/ or Hepatitis a virus/ or Hepatitis a virus, human/ or (Hepatitis adj (a or b or c or e)).ti,ab. (174316)

24 (Coxiella burnetii or Salmonella typhi or Salmonella paratyphi or Burkholderia pseudomallei or Pseudomonas pseudomallei or Brucella or Escherichia coli or E-coli or E coli or Citrobacter freundii or Listeria monocytogenes or (Leptospira adj (kmetyi or interogans or weilii or parva)) or Rickettsia or Orientia tsutsugamushi or mycobacterium tuberculosis or h1n1 or Human Herpesvirus 4 or Human Herpes virus 4 or Ehrlichia or Anaplasma or Bartonella or Borrelia or Neorickettsia or Sennetsu or Blastomyces or Cryptococcus or Coccidioides or Histoplasma or Penicillium marneffii or Talaromyces or Yersinia pestis or Francisella tularensis or Klebsiella pneumoniae or Mycoplasma).ti,ab. (392875)

25 (Q fever or salmonellosis or salmonella infection* or melioidosis or brucellosis or listeria infection* or listeriosis or leptospira* infection* or leptospirosis or rickettsia* infection* or rickettsios* or enteric fever or typhoid fever or paratyphoid fever or plague or anaplasmosis or ehrlichiosis or cat scratch fever or cat scratch disease or trench fever or Carrion* disease or borreliosis or lyme or bartonellosis or relapsing fever or typhus or blastomycosis or cryptococcal or coccidioidomycosis or valley fever or histoplasmosis or penicilliosis or tularaemia or tularemia).ti,ab. (79926)

26 Blood-Borne Pathogens/ (2796)

27 ((gram-positive adj3 (bacteria* or infection*)) or (gram-negative adj3 (bacteria* or infection*))).ti,ab. (41914)

28 Cerebrospinal Fluid/ or (cerebrospinal fluid or csf).ti,ab. (125993)

29 exp Bacterial Infections/ not exp Bacteremia/ (745719)

30 exp Bacteria/ (1126988)

31 or/6-30 (2184417)

32 exp Animals/ (18313008)

33 Humans/ (14228720)

34 32 not (32 and 33) (4084288)

35 (Afghanistan or Bangladesh or Nepal or India or Inde or Pakistan or Bhutan or Bhoutan or Sri Lanka or Maldives).ti,ab. (90852)

36 Afghanistan/ or Bangladesh/ or Nepal/ or exp India/ or Pakistan/ or Bhutan/ or Sri Lanka/ or Maldives/ (109593)

37 or/35-36 (144110) Page 3

38 5 and 31 and 37 (4054)

39 4 and 37 (4197)

40 38 or 39 (7715)

41 40 not 34 (7458)

42 limit 41 to (chinese or english or french or portuguese or spanish) (7306)

43 limit 42 to yr="1980 -Current" (6932)

### **1.2 PRISMA flow-diagram for sub-regions**

**PRISMA flow diagram for Southern Asia**

Figure 1: PRISMA flowchart for the literature search for Southern Asia

**PRISMA flow diagram for South-eastern Asia**

Figure 2: PRISMA flowchart for the literature search for South-eastern Asia

### **1.3 Additional results**

**Table 1: Distribution of articles by age categories**

| **Age group** | **Number of articles** | **Column percentage** |
| --- | --- | --- |
| Neonates | 176 | 7.3% |
| Infants | 47 | 2.0% |
| Children | 408 | 16.9% |
| Adults | 764 | 31.7% |
| All Ages | 757 | 31.4% |
| Age Unspecified | 258 | 10.7% |
| Total | 2,410 |  |

**Table 2: Distribution of articles by sample specimen analysed**

| **Specimen analysed** | **Number of articles** | **Column percentage** |
| --- | --- | --- |
| Blood | 2,068 | 85.8% |
| CSF | 101 | 4.2% |
| Blood or CSF | 60 | 2.5% |
| Bone Marrow or joint or lymph or liver aspirates | 63 | 2.6% |
| Peritoneal or pericardial or pleural fluid | 23 | 1.0% |
| Spleen | 10 | 0.4% |
| Vitreous humor | 10 | 0.4% |
| Not specified | 2 | 0.1% |
| Multiple specimens analysed | 73 | 3.0% |
| Total | 2,410 |  |

**Table 3: Distribution of articles by reported pathogen groups**

| **Pathogen group** | **Number of articles** | **Percentage** |
| --- | --- | --- |
| Only one pathogen group reported  (2,267 articles) |  |  |
| Bacteria | 1235 | 51.2% |
| Viruses | 846 | 35.1% |
| Parasites | 132 | 5.5% |
| Fungi | 54 | 2.2% |
| Multiple pathogens reported  (143 articles) |  |  |
| Bacteria and fungi | 84 | 3.5% |
| Bacteria and viruses | 49 | 2.0% |
| Bacteria and parasites | 4 | 0.2% |
| Bacteria and fungi and viruses | 5 | 0.2% |
| Parasites and viruses | 1 | 0.1% |
| Total Ϯ | 2410 |  |

Ϯ A total of 1,377 articles reported bacteria, 901 reported viruses, 143 reported fungi and 137 reported parasites

| **Diagnostic categories** | **Bacteria** | **Viruses** | **Parasites** | **Fungi** |
| --- | --- | --- | --- | --- |
| Culture | 66.8% (920/1377) | 1.6% (14/901) | 11.7% (16/137) | 87.4% (125/143) |
| Microscopy/staining | 0.5% (7/1377) | 0% (0/901) | 51.1% (70/137) | 2.8% (4/143) |
| PCR | 2.2% (30/1377) | 10% (90/901) | 3.6% (5/137) | 0% (0/143) |
| Serological | 24.7% (340/1377) | 75.5% (680/901) | 28.5% (39/137) | 5.6% (8/143) |
| Multiple diagnostic methods | 5.8% (80/1377) | 13% (117/901) | 5.1% (7/137) | 4.2% (6/143) |
| Total articles Ϯ | 1377 | 901 | 137 | 143 |

Ϯ the number of articles are unique to each pathogen group but not in overall as some articles reported pathogens from multiple groups (e.g. both viruses and parasites)


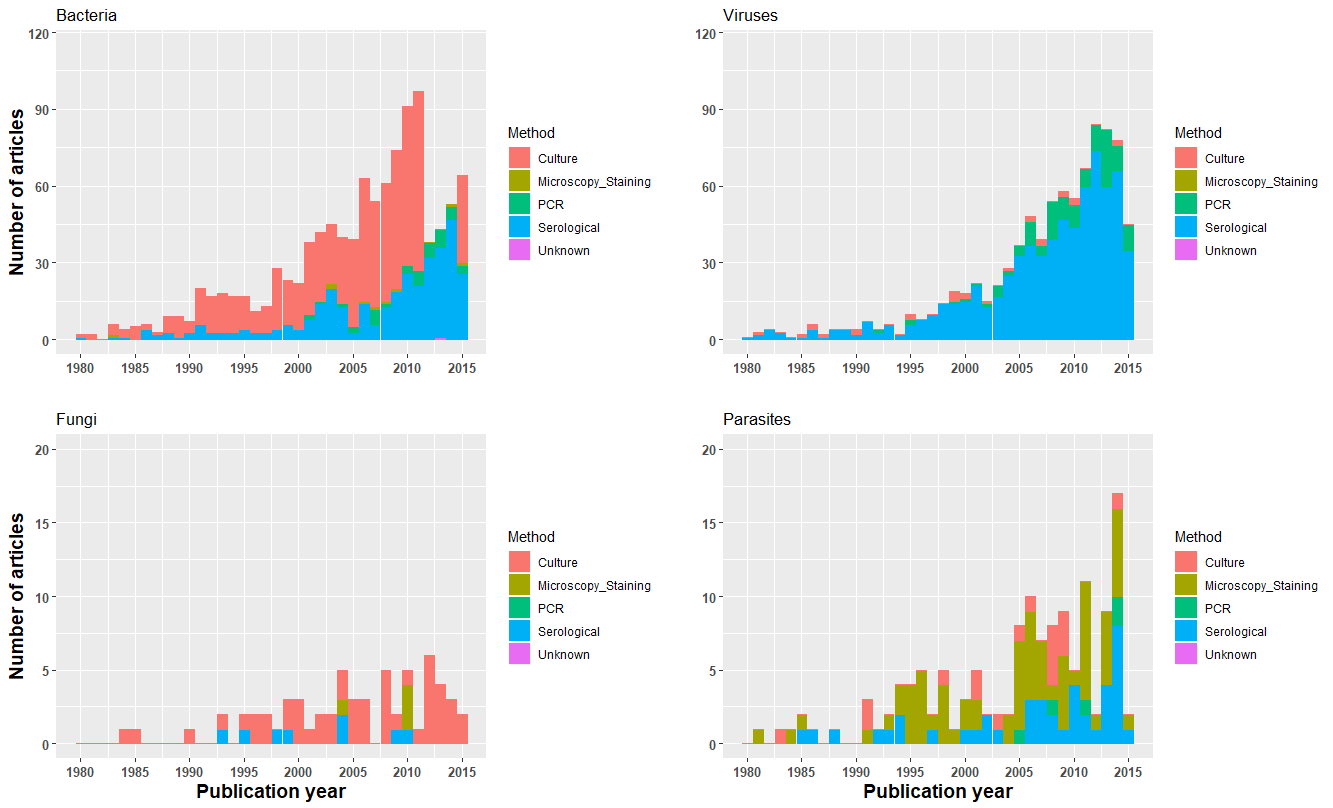


**Figure 1: Diagnostic methods used for identification of pathogens by publication year**

**
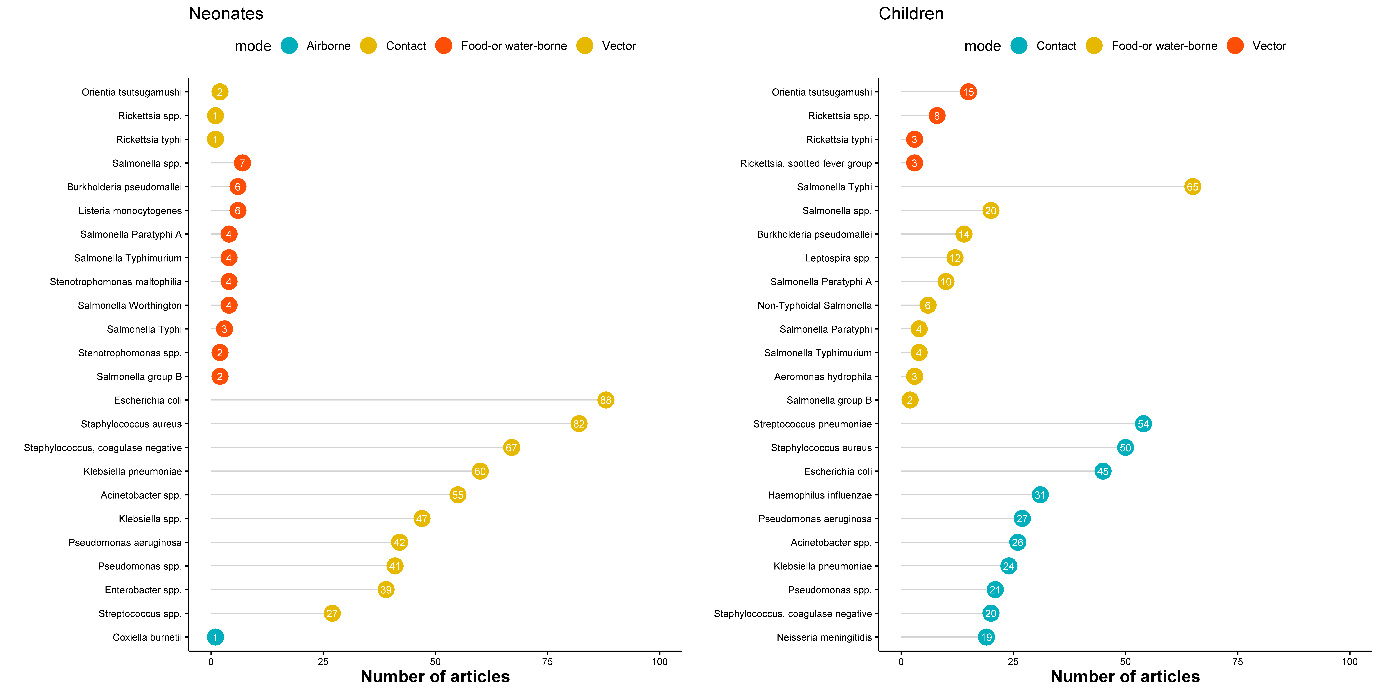
**

**
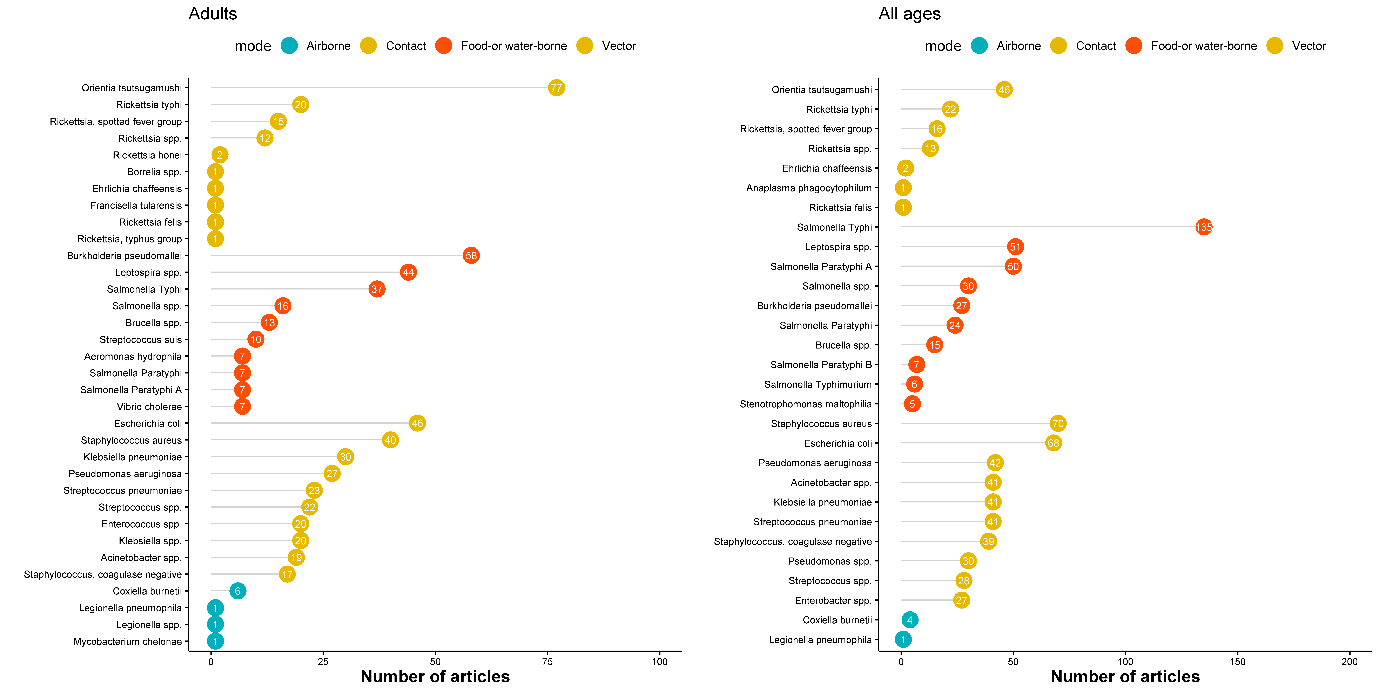
**

**Figure 2: Most commonly reported bacterial infections by mode of transmission and age-categories**

Legend: The graph presents the commonly reported bacterial infections (based on the number of the published articles) by epidemiological mode of transmission and the different age categories reported between 1980-2015 from Southern Asia and South-eastern Asia.


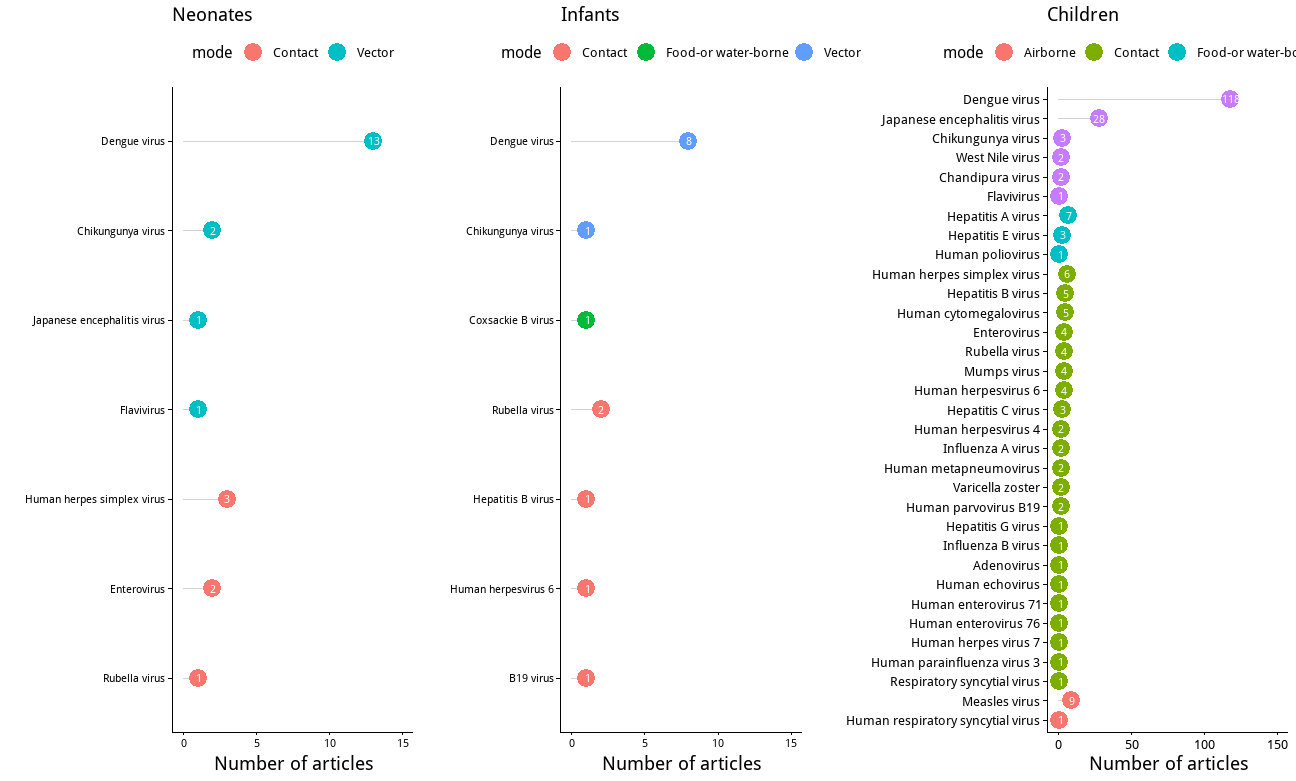


**Figure 3a: Most commonly reported viral infections by mode of transmission for neonates, infants and children**

Legend: The graph presents the common viral infections (based on the number of the published articles) by epidemiological mode of transmission in the neonates, infants and children age categories, reported between 1980-2015 from Southern Asia and South-eastern Asia.


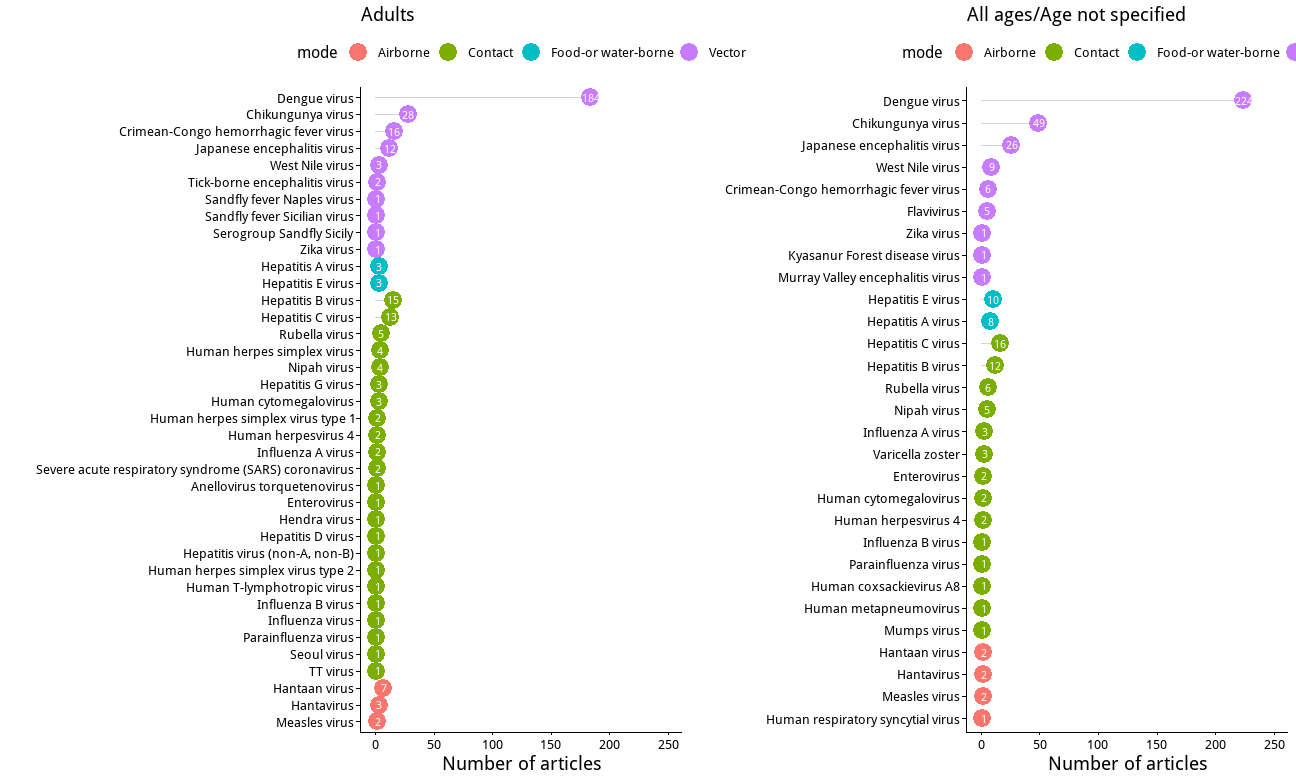


**Figure 3b: Most commonly reported viral infections by mode of transmission for adults and unspecified age-groups**

Legend: The graph presents the common viral infections (based on the number of the published articles) by epidemiological mode of transmission in the adult and the unspecified age group, reported between 1980-2015 from Southern Asia and South-eastern Asia.
